# A National Needs Assessment of Canadian Nurse Practitioners Regarding Cannabis for Therapeutic Purposes

**DOI:** 10.1089/can.2018.0002

**Published:** 2018-03-01

**Authors:** Lynda G. Balneaves, Abeer Alraja, Daniel Ziemianski, Fairleth McCuaig, Mark Ware

**Affiliations:** ^1^ College of Nursing, Rady Faculty of Health Sciences, University of Manitoba, Winnipeg, Canada.; ^2^ Manitoba Centre for Nursing and Health Research, University of Manitoba, Winnipeg, Canada.; ^3^ Canadian Consortium for the Investigation of Cannabinoids, Montreal, Canada.; ^4^ School of Physical and Occupational Therapy, McGill University, Montreal, Canada.; ^5^ School of Nursing, Faculty of Applied Science, University of British Columbia, Vancouver, Canada.; ^6^ Department of Anesthesia, McGill University, Montreal, Canada.; ^7^ Department of Family Medicine, McGill University, Montreal, Canada.; ^8^ Alan Edwards Pain Management Unit, McGill University, Montreal, Canada.

**Keywords:** cannabis, continuing education, health professionals, medical marijuana, needs assessment, nursing

## Abstract

**Introduction:** In Canada, the Access to Cannabis for Medical Purposes Regulations (ACMPR) has given nurse practitioners (NPs) the power to authorize cannabis for therapeutic purposes (CTP) to eligible patients. This expansion in NPs' scope of practice underscores the importance of delivering balanced, evidence-based education on cannabis to NPs. The aim of this national study was to assess NPs' knowledge and practice gaps related to CTP to inform the development of future education resources that increase NPs' clinical competence and improve patient care related to medical cannabis.

**Methods:** This is a quantitative, descriptive exploratory design study. A national online survey of NPs was conducted from August 2013 to June 2014. NPs were recruited through email lists held by numerous Canadian nursing organizations. The survey was adapted from a previous national survey that assessed CTP educational needs among Canadian physicians. The survey assessed NPs' knowledge, experience, barriers, and attitudes related to CTP as well as preferred format for future CTP education.

**Results:** The sample consisted of 182 NPs from across Canada. The largest knowledge gap was related to dosing and creating effective treatment plans for patients using CTP. The majority of respondents (76.3%) ranked the need for education on CTP to be either strong or very strong. Over half (57%) reported that they would be comfortable authorizing medical cannabis through the ACMPR; this number increased to 64% if they were to receive appropriate education.

**Conclusion:** Nursing regulatory organizations, in partnership with academic institutions and government agencies, must work toward the development of educational and clinical competencies specific to CTP. Tailored education programs are needed to address the knowledge gaps held by NPs and the clinical barriers they face to including CTP as part of their care.

## Introduction

In Canada, a national program for access to medical cannabis began in 2001 as the Marihuana Medical Access Regulations (MMAR). This program was in response to public demand and legal decisions requiring the development of a regulatory approach allowing eligible patients to access and possess cannabis for therapeutic purposes (CTP). Since 2001, there have been numerous court challenges and regulatory changes, culminating in the Access to Cannabis for Medical Purposes Regulations (ACMPR), which came into effect in August 2016. The current regulations allow healthcare practitioners (HCPs; i.e., physicians or nurse practitioners [NPs]) to authorize eligible patients to access, produce, and possess CTP.^1^

The ACMPR extended CTP authorization privileges to NPs. Previously, physicians were the only HCPs with the authority to sign a patient's application to possess CTP. This issue affects all Canadian NPs by positioning them alongside physicians as “gatekeepers” for patients' legal access to CTP. The emergence of new CTP regulations creates a need and provides an opportunity for the development and delivery of evidence-based continuing education for NPs. To be effective, a national implementation strategy will be required, as well as the development of clinical competencies related to cannabis for NPs. The first step in such a process is to conduct a needs assessment.

NPs are registered nurses with advanced education and experience who are trained and qualified to diagnose and treat illnesses, order tests, and prescribe medications.^2^ NPs are situated in community care, long-term care, and hospital and NP-led clinic settings, with some specializing in such fields as primary care, cancer, and mental health.^2^ The number of NPs is increasing in Canada, as is their role in the healthcare system.

Currently, NPs in Canada are authorized to prescribe a restricted list of controlled substances if they have completed an approved education program.^3,4^ Substances that have been excluded are cannabis, heroin, opium, cocaine, and some anabolic steroids. Only the College of Nurses of Ontario has permitted NPs to prescribe cannabis and cannabinoids. Thus, despite the ACMPR allowing NPs to authorize CTP use in Canada and the recent expansion of NPs' prescribing authority related to controlled substances, the majority of nursing regulatory bodies in Canada have been hesitant to include cannabis within NPs' scope of practice.

## Literature Review

Previously, the illegal status of cannabis prevented HCPs from discussing the use of CTP with their patients.^5^ However, recent changes to federal regulations regarding CTP in Canada has allowed NPs to authorize the use of CTP to qualifying patients. These changes affect NPs' educational needs, scopes of practice, and liability issues. Nurses must be informed by up-to-date, pharmacological, physiological, psychological, and legal evidence regarding CTP.^6^ To support NPs in providing comprehensive and safe care and patient education related to CTP, it is essential to assess their knowledge gaps, attitudes, and barriers regarding the use of CTP in clinical practice.

Needs assessment analysis is a step included in the systematic approach of instructional design.^7,8^ The first stage of a cannabis education strategy requires a systematic process for identifying and describing information needs; this can be done by measuring the gaps between the current knowledge of CTP and the desired knowledge level.

Previous studies have investigated American physicians' knowledge, attitudes, and opinions regarding the use of CTP.^9,10^ In Canada, the Canadian Consortium for the Investigation of Cannabinoids (CCIC)^*^ conducted a needs assessment along with a continuing education initiative consisting of 64 live events in 26 locations in 2009–2010, with 526 HCPs completing the survey.^11^ The largest gaps in knowledge for physicians (*n*=417) were related to the interactions between cannabinoids and opioids. Physicians also desired knowledge about the long-term risks of cannabinoid use and demonstrated limited understanding of current CTP regulations in Canada. Practice guidelines, clinical data, and long-term safety data were needed in order for physicians to feel more comfortable in authorizing CTP.

An online national survey, adapted from the CCIC needs assessment, was conducted with 426 Canadian physicians to verify their educational needs regarding CTP.^12^ The most desired knowledge was related to “potential risks of using CTP” and “safety, warning signs and precautions for patients using CTP.” The largest gap between perceived current and desired knowledge levels was “dosing” and “the development of treatment plans.” The preferred educational approaches were peer-reviewed literature reviews on specific topics, online learning programs as part of continuing medical education, and online resources.

Only one survey on NPs' CTP knowledge and experiences has been conducted. The College of Registered Nurses of British Columbia gathered input from 157 NPs regarding the expansion of their prescribing rights to include controlled substances.^13^ The majority (86.0%) of NPs anticipated incorporating controlled substances prescribing into their clinical practice. An overwhelming majority (92%), however, perceived themselves to require further education. Regarding CTP, only 31.0% indicated that they would include the authorization of medical cannabis in their practice, if given the authority. Over a quarter of the sample (28.7%) were unwilling to authorize CTP and 40.0% were uncertain about including CTP authorization within their practice.^13^

While these findings capture the experience and attitudes of NPs in British Columbia about CTP, the applicability to NPs in other regions of Canada is uncertain. As Canadian NPs' prescribing authority expands to include CTP, it will be important to understand at a national level existing gaps in knowledge as well as current attitudes and practices within the NP community. The aim of this study was to assess NPs' knowledge and practice gaps related to CTP to inform the development of education resources and to increase clinical competency in the management of cannabis and cannabinoids and improve patient care. Specific research objectives include identifying NPs':

(1) Knowledge gaps concerning the use of CTP
(2) Experiences with cannabinoids and CTP in clinical practice
(3) Attitudes toward cannabinoids and CTP
(4) Barriers to using CTP as a treatment option in clinical practice
(5) Preferred means of education about CTP.

## Methods

The study used a quantitative, descriptive exploratory design. A national online survey of NPs was conducted from August 2013 to June 2014. A convenience sample of NPs were recruited through email lists of nursing organizations, including provincial/territorial colleges of nursing, provincial NP associations, and NPs on the CCIC national mailing list.

The survey was adapted from the national survey developed and used by Ziemianski et al.^12^ The survey consisted of six sections. The first section assessed NPs' current and desired level of CTP knowledge using nine items on a 5-point Likert scale (1—very poor; 5—very good). One item was included that ranked NPs' desire for CTP education on a 5-point Likert scale (1—not at all; 5—very strongly). Overall, this subscale had a Cronbach's alpha of .92. The second section included seven items that assessed NPs' clinical experience related to CTP, using a dichotomous response format (yes/no). The third section assessed clinical barriers to prescribing and providing care related to CTP, with NPs selecting all that applied from a list of 12 potential barriers. A Cronbach's alpha of .72 was reported for this subscale. The fourth section assessed NPs' attitudes regarding which HCPs should be allowed to authorize CTP use, using a dichotomous response format (yes/no; Cronbach's alpha=.70). In addition, four items were used to assess NPs' comfort in addressing CTP as part of their clinical practice using a 5-point Likert scale (1—strongly agree; 5—strongly disagree; Cronbach's alpha=.82). The fifth section assessed NPs' preferred format for future CTP education, with NPs selecting from 11 educational approaches. A final section requested demographic and clinical practice information. The survey took 10–15 min to complete and was conducted using the online survey software, LimeSurvey^©^. Ethics approval was obtained from Genetics/Population Research/Investigator Initiated Studies (GEN) Research Ethics Board at the McGill University Health Centre (No. 13-164 GEN).

Descriptive statistics summarized respondents' demographic information, knowledge, experiences, barriers, attitudes, and preferred educational approaches. Data were entered and analyzed using Microsoft Excel^®^ (Redmond, WA). Perceived knowledge gap was calculated by computing the difference between the current and desired knowledge levels. Rather than using averages, the knowledge gap was calculated based on how much greater the individual's desired knowledge level was compared to their current knowledge level.^14,15^ Only response pairs (i.e., current and desired knowledge) were used for the calculation, and responses, where the indicated desired level was lower than the current level, were also excluded.

## Results

### Participants' demographics

In 2013, there were 3,655 NPs eligible to practice in Canada.^16^ For this study, letters of invitation were emailed to 552 NPs across Canada using existing databases. A total of 227 NPs accessed the national survey, with 182 providing complete data for analysis, resulting in a 33% response rate. Geographically, the participants were from Quebec (26.9%), the Prairie Provinces (25.3%), British Columbia (13.7%), and Ontario (11.0%). The majority completed the survey in English (75.3%), with 52.7% practicing in urban settings. Just over half of the respondents reported having six or more years of experience as an NP (51.1%), with most specializing in family practice (45.1%) or primary care (40.1%). See Table 1 for further details.

**Table 1.** **Participant Demographic Characteristics (*N*=182)**

Characteristics	*n* (%)
Language of completion	
English	137 (75.3)
French	45 (24.7)
Years in practice	
0–5	87 (47.8)
6–10	70 (38.5)
11–15	20 (11.0)
16–20	2 (1.1)
21 or more	1 (0.5)
Missing	2 (1.1)
Practice setting	
Urban	96 (52.7)
Rural	54 (29.7)
Both	30 (16.5)
Missing	2 (1.1)
Practice region	
Quebec	49 (26.9)
Prairies	46 (25.3)
Atlantic	41 (22.5)
British Columbia	25 (13.7)
Ontario	20 (11.0)
Missing	1 (0.5)
Specialization/focus of practice^a^	
Family practice	82 (45.1)
Primary care	73 (40.1)
Cardiology	13 (7.1)
Emergency	13 (7.1)
Mental health	12 (6.6)
Palliative care	12 (6.6)
Nephrology	12 (6.6)
Cancer care	5 (2.7)
Substance use/addiction	3 (1.6)
Other	38 (20.9)

^a^ Participants could select more than one response.

### Knowledge about CTP

Respondents were most knowledgeable about the potential uses (2.57/5.0) and risks (2.39/5.0) of CTP as well as the safety, warning signs, and precautions associated with CTP (2.21/5.0). In contrast, the lowest mean knowledge level was for dosing and creating effective treatment plans related to CTP (1.63/5.0), similarities and differences across cannabis products (1.83/5.0), and the current federal CTP regulations^†^ (1.88/5.0).

The top three ranked knowledge gaps (i.e., desired knowledge level—current knowledge level) were as follows: dosing and creating effective treatment plans for patients using CTP; Health Canada's Marihuana for Medical Purposes (MMPR); and similarities and differences among dried cannabis, other forms of cannabis products, and prescription cannabinoid medications. It is interesting to note that the mean desired knowledge score on all knowledge items were more than 4, indicating high interest in learning more about CTP. The majority of respondents (76.3%) also ranked the need for education on CTP to be either strong or very strong. See Table 2 for further details.

**Table 2.** **Analysis of Knowledge Scores and Gaps for Cannabis for Therapeutic Purposes (Ranked by Gap Size)**

Knowledge items	Mean current knowledge score (1–5)	Mean desired knowledge score (1–5)	Gap^a^
Dosing and creating effective treatment plans for patients using CTP	1.63	4.10	2.60
Health Canada's MMPR	1.88	4.20	2.43
Similarities and differences between dried cannabis, other forms of cannabis products, and prescription cannabinoid medications	1.83	4.14	2.41
Laws and regulations surrounding CTP	2.06	4.21	2.29
Mechanism of action of cannabis/endocannabinoid system	2.15	4.16	2.24
Safety, warning signs and precautions for patients using CTP	2.21	4.22	2.18
Alternative routes of administration of CTP	2.10	4.13	2.11
Potential risks of using CTP	2.39	4.20	1.96
Potential uses of CTP	2.57	4.13	1.79

^a^ Gap was calculated (using individual response pairs)=(desired knowledge level − current knowledge level).

CTP, cannabis for therapeutic purposes; MMPR, Marihuana for Medical Purposes Regulations.

### Experiences with CTP

Close to 60% of respondents declared being approached by a patient or family member to discuss CTP. Only 25.3%, however, reported initiating a discussion about CTP with patients and families in their practice. Almost half of the sample (46.2%) disclosed that they have a patient in their practice who was currently using CTP. With regard to NPs' comfort in prescribing a pharmaceutical form of cannabis, more than half (58.2%) were comfortable in supporting patient's access to cannabinoids in this form.

### Barriers to the use of CTP

The lack of knowledge, education or information regarding CTP was considered to be a barrier to the use of cannabis in clinical practice by the majority of NPs (87.4%). Moreover, the lack of clinical guidelines and insufficient information regarding the appropriate use of CTP were perceived as barriers by 70.3% and 63.7% of respondents, respectively. The complete list of barriers is presented in Table 3.

**Table 3.** **Perceived Barriers to the Use of Cannabis for Therapeutic Purposes (*N*=182)**

Perceived barriers	*n*^a^ (%)
Lack of personal knowledge/education or information regarding the use of CTP	159 (87.4)
Lack of clinical guidelines for the use of CTP	128 (70.3)
Insufficient information regarding the appropriate use of CTP	116 (63.7)
Uncertainty about possible interactions with other medications	110 (60.4)
Concern that patients who request CTP may actually want it for recreational purposes	110 (60.4)
Risks and benefits are not sufficiently clear for potential therapeutic uses	103 (56.6)
Potential liability concerns	95 (52.2)
Instruction from nurse practitioner, nursing or medical associations or licensing bodies	93 (51.1)
Concern about possible side effects	83 (45.6)
Uncertainty over whether cannabis has any therapeutic value	51 (28.0)
Availability of prescription cannabinoids (e.g., Sativex^®^, Marinol^®^ or Cesamet^®^)	37 (20.3)
Belief that cannabis is not an appropriate treatment in a specific case	25 (13.7)
Other	24 (13.2)

^a^ Respondents could select more than one response.

CTP, cannabis for therapeutic purposes.

### Attitudes regarding the CTP

Most of the respondents indicated that specialists (97.3%) and family physicians (86.6%) should be authorized to approve the use of CTP. A proportion of respondents did express the belief that NPs should not be authorized to approve the use of CTP (19.2%). There was also a lack of support for other HCPs to engage in the authorization of CTP. See Table 4 for more information.

**Table 4.** **Beliefs Regarding Who Should Prescribe/Authorize Cannabis for Therapeutic Purposes (*N*=182)**

Healthcare professional	Yes *n* (%)^a^	No *n* (%)^a^
Specialist physicians	177 (97.3)	4 (2.2)
Primary care physicians/family physicians	158 (86.8)	22 (12.1)
Nurse practitioners	144 (79.1)	35 (19.2)
Pharmacists	46 (25.3)	110 (60.4)
Naturopathic doctors	41 (22.5)	112 (61.5)
Traditional Chinese Medicine practitioners	30 (16.5)	120 (65.9)
Registered nurses	8 (4.4)	143 (78.6)

^a^ Percentages may not add up to 100% due to missing data.

The vast majority of NPs surveyed agreed that additional education would make them feel more comfortable discussing CTP (90.7%) and more prepared to provide care to patients using CTP (87.4%). More than 80% agreed that availability of specific training and liability protection would increase their level of comfort with the use of CTP within their clinical practice. See Table 5 for additional information.

**Table 5.** **Factors Impacting Nurse Practitioners' Comfort with Cannabis for Therapeutic Purposes (*N*=182)**

Items	Agree^a^*n* (%)^b^	Neutral *n* (%)^b^	Disagree^a^ I (%)^b^
I would feel more comfortable discussing the use of CTP with patients/patient family members if I had more education about it.	165 (90.7)	11 (6.0)	5 (2.7)
I feel that with more education I would be better able to treat patients using CTP.	159 (87.4)	17 (9.3)	5 (2.7)
I would feel more comfortable if nurse practitioners who participated in the MMPR were required to undergo a specific training or licensing program.	154 (84.6)	17 (9.3)	10 (5.5)
I would feel more comfortable authorizing CTP if Health Canada offered me protection from liability.	147 (80.0)	26 (14.3)	8 (4.4)

^a^ Likert scale responses were collapsed to dichotomous outcomes: Agree (strongly agree and agree) and disagree (strongly disagree and disagree).

^b^ Percentages may not add up to 100% as missing data or nonresponses are not included.

### Education preferences

The most preferred sources of education related to CTP were as follows: online learning programs as part of continuing professional development (76.9%); online resources (64.8%); and workshops/small-group learning sessions (63.2%). See Table 6 for further details.

**Table 6.** **Preferred Method of Receiving Further Educational Information (*N*=182)**

Resource	*n*^a^ (%)
Online learning programs as part of CPD	140 (76.9)
Online resources	118 (64.8)
Workshops/small-group learning sessions	115 (63.2)
Symposia/conferences	107 (58.8)
A monograph on cannabis (similar to a drug product monograph)	95 (52.2)
Peer-reviewed literature reviews on specific topics	95 (52.2)
Expert speaker tour	88 (48.4)
Mentorship/preceptorship program	69 (37.9)
Topic-specific reports	58 (31.9)
Grand rounds	47 (25.8)
Newsletter	35 (19.2)
Other	5 (2.7)

^a^ Respondents could select more than one response.

CPD, Continuing Professional Development.

## Discussion

As interest in CTP grows in North America, HCPs will be faced with increasing requests for information and authorization from patients. With federal regulations in Canada allowing NPs to authorize and assist patients in the administration of CTP, and with continued reluctance by Canadian physician groups to engage in care involving CTP,^17,18^ NPs may become a major source of education, decision support, and access for patients. This is the first study in Canada that examines the knowledge and practice gaps related to CTP in a national sample of NPs, and the findings provide direction regarding future education programming.

NPs in this study perceived themselves to have a low level of knowledge about CTP. Specifically, they lacked knowledge regarding clinical recommendations (i.e., dosing, strains, products, and care plans), current CTP regulations, the potential risks and benefits of CTP, as well as the endocannabinoid system and mechanisms of action of cannabinoids. NPs expressed a strong need for education on all aspects of CTP.

The largest knowledge gaps identified were related to clinical practice issues associated with CTP, including dosing protocols, effective treatment plans, and the use of different forms of cannabis products. These gaps were comparable to those identified by physicians,^12^ highlighting the current lack of evidence-based clinical practice guidelines related to CTP.^19,20^ Similar to physicians,^12^ NPs perceived themselves to lack knowledge about current federal CTP regulations. Given the significant role NPs were given in the ACMPR with regard to providing authorization for CTP, this lack of knowledge is striking and raises concerns regarding the lack of communication between the nursing community in Canada and federal policymakers responsible for CTP regulations. This finding may also reflect the limited role that NPs have been given, to date, in authorizing and supporting patients in the administration of CTP by their professional regulatory organizations.

Despite more than half of the respondents being approached to discuss the use of CTP, only a quarter reported initiating a discussion about CTP with patients and families in their practice. This may reflect NPs' perceived lack of knowledge regarding CTP, which was identified by respondents as a major barrier to providing care, and aligns with previous research that has shown the majority of HCPs feel unprepared to advise patients about CTP.^21^ NPs' hesitancy may also be a consequence of their need to be accountable for their practice—without clinical practice guidelines and practice standards specific to CTP, NPs may feel uncertain about how to appropriately advise patients about the potential risks and benefits of CTP and may be concerned about liability issues. Fear of diversion of CTP for recreational purposes was also a substantial concern, which may reflect the long-standing stigma that has been associated with cannabis as a controlled substance in Canada^22^ and the high prevalence of recreational cannabis use among Canadians.^23^ Impending legalization of cannabis in Canada may alleviate this concern among NPs and allow consultations about the therapeutic potential of cannabis to occur.

A surprising number of respondents were unsupportive of NPs having the ability to authorize patients' access to CTP. This finding may again reflect NPs' concerns about the lack of evidence-based information and guidelines related to CTP and their legal standing in supporting CTP use in clinical and community settings. This hesitation, however, may also reflect some NPs' unwillingness to change their clinical practice and accept responsibility for patients using controlled substances, such as cannabis.^24^ Nationally, only a quarter of physicians expressed support for NPs to authorize the use of CTP,^12^ which raises the specter of possible interprofessional conflict, especially as NPs' prescriptive authority expands across Canada to include cannabis.

The findings must be considered with caution given the small sample size, which represented only a small percentage of the NP workforce in Canada. As a convenience sample, NPs who participated in the survey may have also held a unique perspective on CTP that was not representative of the larger NP community. The sample, however, was regionally diverse and included NPs from a variety of practice settings. It is important to note that the survey occurred before the ACMPR coming into effect and the Canadian government's announcement regarding legalization of non-medical cannabis. Given the limited movement on NPs' prescribing authority related to CTP by nursing regulatory bodies following implementation of the ACMPR, the findings remain relevant and will inform future education programming.

## Implications

As the evidence and demand for CTP increases, education within NP programs as well as continuing education for those in practice is urgently needed that covers content that spans from the endocannabinoid system, to potential risks and benefits across diverse health conditions and populations, to clinical care decisions related to dosage, strain, route of administration, and product.^25^ In addition, the development of tailored education that addresses CTP in the context of NPs' scope of practice and regulatory issues, and the transition in prescriptive authority in nursing, would enhance not only NPs' knowledge but also potentially their ability to navigate the barriers surrounding their engagement in CTP authorization and care. A diversity of educational strategies, including online continuing professional development and in-person seminars, are required to best meet the diverse educational needs of NPs. While online courses on controlled substances are available for NPs in Canada (e.g., College of Nurses of Ontario^26^), these courses are not specific to CTP and may not fully address the complexities of cannabis use and the rapidly developing body of evidence.

There is an obvious need for clinical research on CTP to inform the development of clinical practice guidelines that will support all HCPs in providing effective and safe care related to cannabis. Several research priority setting meetings have been held in Canada, which have repeatedly called for clinical studies exploring dosing, product and administration protocols for CTP, as well as basic science research on the endocannabinoid system.^27^ Research is also needed that explores in more depth NPs' attitudes and beliefs related to CTP and the effectiveness of different education strategies on NP practice related to CTP. Finally, exploration of the institutional and societal barriers to the expansion of NPs' scope of practice is essential to empower NPs to take a leading role in health services related to CTP.

## Conclusion

To support NPs in fulfilling their federally legislated role in authorizing and providing safe care to patients who use CTP, NPs will need appropriate educational preparation to expand their scope of practice. Nursing regulatory organizations, in partnership with academic institutions and government agencies, must work toward the development of educational and clinical competencies specific to CTP. Tailored education programs are needed to address the knowledge gaps held by NPs and the clinical barriers they face to including CTP as part of their practice.

## Abbreviations Used

ACMPR: Access to Cannabis for Medical Purposes Regulations
CCIC: Canadian Consortium for the Investigation of Cannabinoids
CTP: cannabis for therapeutic purposes
HCPs: healthcare practitioners
NP: nurse practitioner

## Notes

^*^ CCIC is a registered Canadian not-for-profit organization that aims to increase research and education on cannabinoid and endocannabinoid agents.

^†^ During the survey, the MMPR were in effect.

## References

[1] Minister of Justice. Access to cannabis for medical purposes regulations. 2016 www.laws-lois.justice.gc.ca/PDF/SOR-2016-230.pdf (last accessed 1219, 2017)

[2] Canadian Nurse Association. Collaborative integration plan for the role of nurse practitioners in Canada. 2011 www.cna-aiic.ca (last accessed 1219, 2017)

[3] Canadian Nurses Association. Health Canada grants nurse practitioners more prescribing authority. 2012 www.cna-aiic.ca/en/news-room/news-releases/2012/health-canada-grants-nurse-practitioners-more-prescribing-authority (last accessed 710, 2017)

[4] Minister of Justice. New classes of practitioners regulations. 2012 www.laws-lois.justice.gc.ca/PDF/SOR-2012-230.pdf (last accessed 1219, 2017)

[5] Mathre ML Policy perspectives: therapeutic cannabis. Am J Nurs. 2001;101:61–68 10.1097/00000446-200104000-00026 11301687

[6] Green AJ, De-Vries K Cannabis use in palliative care: an examination of the evidence and the implications for nurses. J Clin Nurs. 2010;19:2454–2462 20920073 10.1111/j.1365-2702.2010.03274.x

[7] Gustafson RM, Branch KL Survey of instructional development models. Information Resources Publications: Syracuse, NY, 1997

[8] Altschuld JW, Witkin BR From needs assessment to action: transforming needs into solution strategies. Sage Publications: Thousand Oaks, CA, 2000

[9] Charuvastra A, Friedmann PD, Stein MD Physician attitudes regarding the prescription of medical marijuana. J Addict Dis. 2005;24:87–93 16186085 10.1300/J069v24n03_07

[10] Kondrad E, Reid A Colorado family physicians' attitudes toward medical marijuana. J Am Board Fam Med. 2013;26:52–60 23288281 10.3122/jabfm.2013.01.120089

[11] Ziemiansk D, Tekanoff R, Luconi F, et al. Cannabinoids in clinical practice: experiences and educational needs. In Conference: Canadian Pain Society, Montreal, 2012 www.researchgate.net/publication/260476224_CANNABINOIDS_IN_CLINICAL_PRACTICE_EXPERIENCES_AND_EDUCATIONAL_NEEDS_Daniel_Ziemianski_BSc_Canadian_Consortium_for_the_Investigation_of_Cannabinoids_Rory_Tekanoff_MSc_RxMedia_Inc_Mississauga_Ontario_Fr (last accessed 1219, 2017)

[12] Ziemianski D, Capler R, Tekanoff R, et al. Cannabis in medicine: a national educational needs assessment among Canadian physicians. BMC Med Educ. 2015;15:1–7 25888752 10.1186/s12909-015-0335-0 PMC4374299

[13] College of Registered Nurses of British Columbia. Nurse practitioner controlled substances post survey analysis. 2013 www.crnbc.ca/crnbc/Documents/2013_06_27_NP_Controlled_Substances_Survey_Results.pdf (last accessed 1219, 2017)

[14] Fox RD Revisiting discrepancy analysis in continuing medical education: a conceptual model. J Contin Educ Health Prof. 2011;31:71–76 21425364 10.1002/chp.20104

[15] Moore DE, Green JS, Gallis HA Achieving desired results and improved outcomes: integrating planning and assessment throughout learning activities. J Contin Educ Health Prof. 2009;29:1–15 19288562 10.1002/chp.20001

[16] Canadian Institute for Health Information. Regulated nurses, 2013. 2014 www.secure.cihi.ca/free_products/Nursing-Workforce-2013_EN.pdf (last accessed 1219, 2017)

[17] Omand G Why doctors don't want to be the gatekeepers of medical marijuana. The Canadian Press: Vancouver, 2017

[18] Thompson JW, Koenen MA Physicians as gatekeepers in the use of medical marijuana. J Am Acad Psychiatry Law. 2011;39:460–464 22159973

[19] Chan A, Molloy L, Pertile J, et al. A review for Australian nurses: cannabis use for anti-emesis among terminally ill patients in Australia. Aust J Adv Nurs. 2017;34:43–47

[20] Hall JM, Shattell MM, McConnell EA The marijuana phenomenon: contradictions and silence. J Addict Nurs. 2016;27:1–6 26950836 10.1097/JAN.0000000000000105

[21] Brooks E, Gundersen DC, Flynn E, et al. The clinical implications of legalizing marijuana: are physician and non-physician providers prepared? Addict Behav. 2017;72:1–7 28319813 10.1016/j.addbeh.2017.03.007

[22] Bottorff JL, Bissell LJ, Balneaves LG, et al. Perceptions of cannabis as a stigmatized medicine: a qualitative descriptive study. Harm Reduct J. 2013;10:1–10 23414118 10.1186/1477-7517-10-2 PMC3584982

[23] Rotermann M, Langlois K Prevalence and correlates of marijuana use in Canada, 2012. Health Rep. 2015;26:10–15 25875158

[24] Kaplan L, Brown MA The transition of nurse practitioners to changes in prescriptive authority. J Nurs Scholarsh. 2007;39:184–190 17535320 10.1111/j.1547-5069.2007.00165.x

[25] Ware MA, Ziemianski D Medical education on cannabis and cannabinoids: perspectives, challenges, and opportunities. Clin Pharmacol Ther. 2015;97:548–550 25728558 10.1002/cpt.103

[26] College of Nurses of Ontario. Q&As: controlled substances education requirement. 2017 www.cno.org/en/trending-topics/nps-and-prescribing-controlled-substances/qas-controlled-substances-education-requirement (last accessed 1219, 2017)

[27] The Arthritis Society. Clearing the air: summary report of the medical cannabis research roundtable. 2015 www.arthritis.ca/getmedia/f9e3dc04-b3e9-482e-9109-848590a84b7f/Clearing-the-Air-EN-FINAL.pdf (last accessed 1219, 2017)

